# Supplementary material for: Clinical outcomes associated with prostate cancer conspicuity on biparametric and multiparametric MRI: a protocol for a systematic review and meta-analysis of biochemical recurrence following radical prostatectomy
Source: BMJ Open. 2021 May 5;11(5):e047664. doi: 10.1136/bmjopen-2020-047664 (PMC8103365; doi:10.1136/bmjopen-2020-047664)
Supplement: Author's manuscript [file bmjopen-2020-047664.draft_revisions.pdf]

BMJ Open

BMJ Open is committed to open peer review. As part of this commitment we make the peer review history of every article we publish publicly available.

When an article is published we post the peer reviewers' comments and the authors' responses online. We also post the versions of the paper that were used during peer review. These are the versions that the peer review comments apply to.

The versions of the paper that follow are the versions that were submitted during the peer review process. They are not the versions of record or the final published versions. They should not be cited or distributed as the published version of this manuscript.

BMJ Open is an open access journal and the full, final, typeset and author-corrected version of record of the manuscript is available on our site with no access controls, subscription charges or pay-per-view fees (http://bmjopen.bmj.com).

If you have any questions on BMJ Open's open peer review process please email info.bmjopen@bmj.com

BMJ Open

Clinical outcomes associated with prostate cancer conspicuity on multiparametric magnetic resonance imaging: a protocol for a systematic review and meta-analysis of biochemical recurrence post-radical prostatectomy

Journal:	*BMJ Open*
Manuscript ID	bmjopen-2020-047664
Article Type:	Protocol
Date Submitted by the Author:	08-Dec-2020
Complete List of Authors:	Morka, Naomi; University College London Medical School,<br>Simpson, Benjamin Scott; University College London, UCL Division of Surgery & Interventional Science<br>Ball, Rhys; University College London Hospitals NHS Foundation Trust, Department of Pathology<br>Freeman, Alex; University College Hospital London, Department of Histopathology<br>Kirkham, Alex; University College London Hospitals NHS Foundation Trust, Department of Radiology<br>Kelly, Daniel; Cardiff University, School of Healthcare Sciences, College of Biomedical and Life Sciences<br>Whitaker, Hayley; University College London, UCL Division of Surgery and Interventional Science<br>Emberton, Mark; University College London, Division of Surgery and Interventional Sciences; University College London Hospital, Department of Urology<br>Norris, Joseph; University College London, UCL Division of Surgery & Interventional Science
Keywords:	UROLOGY, Prostate disease < UROLOGY, Urological tumours < UROLOGY, Magnetic resonance imaging < RADIOLOGY & IMAGING

SCHOLARONE™
Manuscripts

I, the Submitting Author has the right to grant and does grant on behalf of all authors of the Work (as defined in the below author licence), an exclusive licence and/or a non-exclusive licence for contributions from authors who are: i) UK Crown employees; ii) where BMJ has agreed a CC-BY licence shall apply, and/or iii) in accordance with the terms applicable for US Federal Government officers or employees acting as part of their official duties; on a worldwide, perpetual, irrevocable, royalty-free basis to BMJ Publishing Group Ltd ("BMJ") its licensees and where the relevant Journal is co-owned by BMJ to the co-owners of the Journal, to publish the Work in this journal and any other BMJ products and to exploit all rights, as set out in our licence.

The Submitting Author accepts and understands that any supply made under these terms is made by BMJ to the Submitting Author unless you are acting as an employee on behalf of your employer or a postgraduate student of an affiliated institution which is paying any applicable article publishing charge ("APC") for Open Access articles. Where the Submitting Author wishes to make the Work available on an Open Access basis (and intends to pay the relevant APC), the terms of reuse of such Open Access shall be governed by a Creative Commons licence – details of these licences and which Creative Commons licence will apply to this Work are set out in our licence referred to above.

Other than as permitted in any relevant BMJ Author's Self Archiving Policies, I confirm this Work has not been accepted for publication elsewhere, is not being considered for publication elsewhere and does not duplicate material already published. I confirm all authors consent to publication of this Work and authorise the granting of this licence.

For peer review only

Clinical outcomes associated with prostate cancer conspicuity on multiparametric magnetic resonance imaging: a protocol for a systematic review and meta-analysis of biochemical recurrence post-radical prostatectomy

Naomi Morka[1], Benjamin S. Simpson[2], Rhys Ball[3], Alex Freeman[3], Alex Kirkham[4], Daniel Kelly[5], Hayley C. Whitaker[2], Mark Emberton,[1,6]* Joseph M. Norris,[2,6,7]

ME and JMN are joint senior authors.

[1] University College London Medical School, London, UK.

[2] UCL Division of Surgery & Interventional Science, University College London, London, UK

[3] Department of Pathology, University College London Hospitals NHS Foundation Trust, London, UK

[4] Department of Radiology, University College London Hospitals NHS Foundation Trust, London, UK

[5] School of Healthcare Sciences, Cardiff University, Wales, UK

[6] London Deanery of Urology, London, UK

[7] Department of Urology, University College London Hospitals NHS Foundation Trust, London, UK

Correspondence to: Joseph M Norris;

UCL Division of Surgery & Interventional Science

43-45 Foley St, Fitzrovia, London. W1W 7TY

joseph.norris@ucl.ac.uk

For peer review only

Funding: Norris is funded by the Medical Research Council (MRC) (MR/S00680X/1). Simpson is funded by the Rosetrees Trust. Emberton receives research support from the United Kingdom's National Institute of Health Research (NIHR) UCLH/UCL Biochemical Research Centre.

Ethics: No ethical considerations apply.

Word count: 1511 words

Conflicts of interest: Norris receives funding from the MRC. Simpson receives funding from the Rosetrees Trust. Whitaker receives funding from the PCUK, the Urology Foundation and Rosetrees Trust. Kirkham, Freeman and Emberton have stock interest in Nuada Medical Ltd. Emberton acts as a consultant, trainer and proctor to Sonatherm Inc; Angiodynamics Inc; Exact Imaging Inc.

For peer review only - http://bmjopen.bmj.com/site/about/guidelines.xhtml

ABSTRACT

Introduction: There is an increasing body of evidence to suggest that visibility of prostate cancer on multiparametric magnetic resonance (mpMRI) may be related to likelihood of adverse pathological outcomes. Biochemical recurrence (BCR) after radical prostatectomy remains a significant clinical challenge and a means of predicting likelihood of this prior to surgery could inform treatment choice. It appears that mpMRI could be a potential candidate strategy for BCR prediction, and as such, there is a need to review extant literature on the prognostic capability of mpMRI. Here, we describe a protocol for a systematic review and meta-analysis of the utility of mpMRI in predicting biochemical recurrence following radical prostatectomy for prostate cancer treatment.

Methods and analysis: PubMed, MEDLINE, Embase and Cochrane databases will be searched and screening will be guided by the preferred reporting items for systematic reviews and meta-analyses (PRISMA) guidelines. In order to meet the inclusion criteria, papers must be English-language articles involving patients who have had mpMRI for suspected prostate cancer and have undergone radical prostatectomy as definitive therapy. Patients must have had prostate specific antigen (PSA) monitoring before and after surgery. All relevant papers published from July 1977 to October 2020 will be eligible for inclusion. The Newcastle-Ottawa score will be used to determine the quality and bias of the studies. This protocol is written in-line with the PRISMA protocol 2015 checklist.

Ethics and dissemination: There are no relevant ethical concerns. Dissemination of this protocol will be via peer-reviewed journals as well as national and international conferences.

PROSPERO registration number CRD42020206074

For peer review only

Strengths and limitations of this study

- This study will be the first comprehensive systematic review and meta-analysis of the utility of mpMRI in the prediction of biochemical recurrence following radical prostatectomy, written in-line with PRISMA guidelines.
- The results of this study will further enrich the existing molecular, genomic and pathological data on the clinical utility of prostate cancer conspicuity on mpMRI.
- There is considerable heterogeneity of the literature in this subject area and this study will highlight this, potentially allowing for a more standardised and uniform approach in subsequent work.

For peer review only - http://bmjopen.bmj.com/site/about/guidelines.xhtml

- However, as a result of this inherent heterogeneity, there may be limitations to the strength of conclusions drawn**.**
- Also, due to the relatively recent introduction of prostatic MRI scans, there limited long term data on the clinical outcomes associated with mpMRI.

For peer review only

BACKGROUND

Prostate cancer is a one of the leading causes of cancer mortality in men worldwide, with over a million new cases diagnosed in 2018 alone[1]. Radical prostatectomy is the most commonly employed definitive treatment for patients with this disease[2], often yielding favourable oncologic outcomes[3]. However, biochemical recurrence (BCR), taken to be a serum prostate specific antigen (PSA) level greater than or equal to 0.2ng/dL on two consecutive occasions[4] occurs in approximately 15-40% of patients[5,6]. Prognostically, BCR has high utility and is associated with increased incidence of metastatic disease[4,7]. It follows then, that a reliable approach to predicting BCR could inform therapeutic decision making[8]. Several models have been proposed for prognostication of BCR following radical prostatectomy[9–12], however, these have been criticised for apparent inaccuracy and inapplicability to certain clinical settings[13,14]. This highlights the need for a widely acceptable and accessible means of understanding prostate cancer prognosis in this patient group.

Multiparametric magnetic resonance imaging (mpMRI) has increasingly become an integral part of prostate cancer screening and diagnosis. In recent years, evidence has emerged on the utility of mpMRI in predicting adverse pathology with MRI-invisible tumours suggesting a good prognosis and MRI-visible tumours associated with unfavourable clinical outcomes such as extracapsular extension and seminal vesicle invasion[15–18]. Given that these pathological factors are also associated with increased likelihood of BCR[9,10], it has been suggested that mpMRI may have a role in the predition of BCR after radical prostatectomy[19,20]. This is also supported by studies that show that the visibility of prostate tumours on mpMRI may corelate with genetic markers of poor clinical outcome[21] . Further exploration of role of mpMRI in predicting BCR after radical prostatectomy is necessary as its use in this way could enhance and improve existing models of prostate cancer prognosis, allowing for better clinical decision making and patient care.

The aim of this systematic review is to evaluate and collate the existing literature on the prognostication of BCR after radical prostatectomy using pre-treatment mpMRI. This will be the first of its kind and it will assess the utility of pre-operative mpMRI as a risk stratification tool and predictor of long term clinical outcomes.

For peer review only - http://bmjopen.bmj.com/site/about/guidelines.xhtml

METHODS AND ANALYSIS

This protocol for this systematic review has been written in compliance with the guidance set out in the Preferred Reporting Items for Systematic Reviews and Meta-analysis Protocol (PRISMA-P) 2015 checklist[22]. Included studies will undergo thorough appraisal and analysis to reveal the extent to which pre-operative mpMRI status results may be indicative of the likelihood to develop biochemical recurrence after radical prostatectomy. This protocol has been registered on the PROSPERO database with the registration number: CRD42020206074.

Search Methodology

Searches will be performed on the PubMed, MEDLINE, Embase and Cochrane databases to identify all potentially relevant studies published from July 1977 (date of first human MRI)[23] to October 2020. Free text searches and Medical Subject Headings (MESH) terms will be used with suitable Boolean operators. The use of search terms such as 'prostate,' 'cancer,' and 'mpMRI,' along with alternative terms for 'biochemical recurrence,' 'radical prostatectomy' and 'prognosis' will ensure a comprehensive approach. Three reviewers will be involved in article selection and evaluation and will employ the use Rayyan, a systematic review collaboration tool to improve ease of collaboration, screening of studies and removal of duplicates. To minimize likelihood of overlooking relevant literature, reference lists for each article will be manually screened and experts in the field will be approached.

Study selection and data extraction

Studies will be screened by three separate reviewers using their title and abstract. Following this, the full text articles will then be scrutinized against the inclusion criteria. Any disagreements will be resolved by consensus. Exclusions will be documented with the reasons for each case, allowing generation of the PRISMA flowchart.

Inclusion criteria

To be selected for this review, studies must involve patients with suspected prostate cancer who have undergone a pre-treatment mpMRI, and both pre- and post-radical prostatectomy serum PSA testing.

For peer review only - http://bmjopen.bmj.com/site/about/guidelines.xhtml

Exclusion criteria

Case reports, expert opinions, conference abstracts, reviews and non-English language articles will be ineligible for inclusion. Studies with patient cohorts who have received treatment prior to surgery will also be excluded.

Data extraction

After selection, all articles will meticulously read and examined, with relevant data points extracted and inputted onto a group spreadsheet. Three or more independent reviewers will confirm the data and check for accuracy. Collation of data will be carried out as described by our group previously[24]. The data to be collected is summarised in Table 1.

Table 1 **Data collection items**

Item number	Data title	Data type
**1**	Year of publication	Study characteristic
**2**	Author names	Study characteristic
**3**	Study design	Study characteristic
**4**	Patient population	Demographics
**5**	Number of patients	Demographics
**6**	Pre-operative PSA level	Demographics
**7**	Pre-operative mpMRI status	Demographics
**8**	Number of years of radiologist experience	Methodology
**9**	mpMRI scoring system	Methodology
**10**	mpMRI scanner power	Methodology
**11**	Definition of tumour visibility	Methodology
**12**	Definition of biochemical recurrence	Methodology
**13**	Total follow up time	Methodology
**14**	Biochemical recurrence status	Outcome
**15**	Hazard ratio of BCR	Outcome

BCR, biochemical recurrence; mpMRI; multiparametric magnetic resonance imaging; PSA, prostate specific antigen.

For peer review only - http://bmjopen.bmj.com/site/about/guidelines.xhtml

Endpoints

The main endpoint of this review will be the identification of significant differences in effect estimates such as hazard ratios for BCR between different mpMRI status groups (e.g. disease visibility or invisibility). Other long-term measures of clinical outcome such as mortality will form secondary endpoints where available. Moderator variables, such as mpMRI scoring systems (e.g. PI-RADS vs Likert) and MRI scanner power will be compared, and any which are of a notable impact to the final summary estimate will be recorded.

Risk of bias in individual studies

The bias and quality of the included studies will be assessed by three independent reviewers using the Newcastle-Ottawa score. The three sections of this system (selection, comparability and outcome) will allow for an evaluation of the methods and assessment of the quality of each study. The outcome of which will aid in the thematic synthesis. Any disagreements between the reviewers will be resolved by consensus. In the case that a study is found to be at risk of significant bias or is of unsatisfactory standard, it may be excluded. If the study is still deemed fit to be included in the analysis, this will be duly noted as a limitation in the discussion and conclusion portions of the systematic review. As previously described by us elsewhere[25], sections of the Newcastle-Ottawa scoring system may be modified to better suit the subject area of this review and ensure a greater accuracy in the findings. Risk of bias score will be assessed as a potential moderator variable in the meta-analysis.

Meta-analysis

The distribution of untransformed, logit and double-arcsine transformed ratios will be compared and will be assessed for normality using density plots and tested using the Shapiro-Wilk test. Whichever set of ratios most resemble a normal distribution will be used for further analysis. Inter-study variation will be quantified using I^2 and if statistically significant, a random-effects model will be fitted for estimation the summary estimate. After fitting a model to all relevant studies, leave-one-out analyses (LOO) and accompanying diagnostic plots would be used to identify influential studies including: externally studentized residuals, difference in fits values (DFFITS), Cook's distances, covariance ratios, LOO estimates of the amount of heterogeneity, LOO values of the test statistics for heterogeneity, hat values and weights. Each study would be removed one at a time, and the summary proportion is re-estimated based on the remaining n-1 studies.

For peer review only - http://bmjopen.bmj.com/site/about/guidelines.xhtml

Studies with a statistically significant influence on the fitted model would be removed as outliers and the model re-fitted. Finally, a summary estimate comprising the remaining studies will be calculated to estimate the true hazard associated with mpMRI visibility or the inverse, a mpMRI invisibility. Sub-group analyses are planned between Likert and PI-RADS scoring systems. Data collection items 1,4,8,9,10,11,12 and 13 will be tested as potential moderators assuming a sufficient number of studies are present within each group. All data analysis and visualisation will be performed using the R statistical environment (version 3.6.1, 2019-07-05) using the “metafor" and "meta” packages.

Patient and public involvement

No patients were involved in this study.

For peer review only

For peer review only - http://bmjopen.bmj.com/site/about/guidelines.xhtml

DISCUSSION

There is an increasing need to risk stratify prostate cancers, to accurately identify disease that is more likely to take an adverse clinical course[1,26]. This systematic review aims to address this by appraising current evidence on whether tumour mpMRI visibility is related to BCR of prostate cancer after radical prostatectomy, therefore evaluating the potential use of mpMRI as a risk stratification tool in this population.

Current evidence suggests an association between visibility of prostate cancer on mpMRI, Gleason grade, tumour size[27,28]. Furthermore, it appears that mpMRI conspicuity is positively correlated with D'Amico risk nomogram scores[29]. In terms of the prognostic potential of mpMRI, it has been demonstrated that predictive power of mpMRI may extend beyond its relationship with histopathological markers of disease progression. Indeed, raised Prostate Imaging-Reporting and Data System (PI-RADS) scores[30] and apparent diffusion coefficient (ADC) values have been shown to independently predict the likelihood of BCR[31,32] .

Although there is a clear potential role for mpMRI in predicting BCR after radical prostatectomy, variations in study methodology, such as biopsy sampling, definitions of mpMRI positivity and clinically significant prostate cancer may prevent formation of robust conclusions. We hope to address the heterogeneity in the literature and encourage a more uniform approach to research in this subject area.

In summary, early evidence suggests that mpMRI could provide a means to predict BCR of prostate cancer after radical prostatectomy. The wide availability of mpMRI and non-invasive nature makes it an ideal candidate for potential BCR risk stratification[33]. Our systematic review will assess and analyse the available evidence, with the aim of providing a holistic view of the literature. This will lead to a clearer understanding of the subject, which could inform the choice of appropriate therapy in patients with prostate cancer.

Trial status

- Preliminary searches: started.
- Piloting of the selection study process: not started.
- Formal screening: not started.
- Data extraction: not started.
- Risk of bias assessment: not started.

For peer review only - http://bmjopen.bmj.com/site/about/guidelines.xhtml

- Data analysis: not started.

Draft of search strategy for MEDLINE, Embase, PubMed and Cochrane Databases: ((prostat* NOT prostatitis) AND ("cancer" OR tumo?r* OR malignancy*)) AND ("magnetic resonance imaging" OR "MRI" OR "mpMRI" OR "multi-parametric magnetic resonance imaging") AND ("biochemical recurrence" OR "BCR" OR "biochemical failure") AND ("radical prostatectomy" OR "surgery") AND (prognos* OR predict*)

ETHICS AND DISSEMINATION

There are no relevant ethical concerns. Dissemination of this protocol will be via peer-reviewed journals as well as national and international conferences.

Author affiliations

[1] University College London Medical School, London, UK.

[2] UCL Division of Surgery & Interventional Science, University College London, London, UK

[3] Department of Pathology, University College London Hospitals NHS Foundation Trust, London, UK

[4] Department of Radiology, University College London Hospitals NHS Foundation Trust, London, UK

[5] School of Healthcare Sciences, Cardiff University, Wales, UK

[6] London Deanery of Urology, London, UK

[7] Department of Urology, University College London Hospitals NHS Foundation Trust, London, UK

Email Naomi Morka: naomi.morka.17@ucl.ac.uk, Benjamin S. Simpson: b.simpson@ucl.ac.uk, Rhys Ball: rhys.ball@nhs.net, Alex Freeman: alex.freeman2@nhs.net, Alex Kirkam: alexkirkham@nhs.net, Daniel Kelly: kellydm@cardiff.ac.uk, Hayley C. Whitaker: hayley.whitaker@ucl.ac.uk, Mark Emberton: m.emberton@ucl.ac.uk, Joseph M. Norris: joseph.norris@ucl.ac.uk

Twitter Naomi Morka @naomi_morka, Benjamin S. Simpson @b3nsimp, Daniel Kelly @profdkelly, Hayley C. Whitaker @HayleyWhitaker, Mark Emberton @EmbertonMark, Joseph M. Norris @MrJosephNorris

Contributors The authors' contribution includes, but is not limited to, the following: NM, BSS and JMN drafted the manuscript and created the study concept. RB, AK, AF, DK, HCW and ME provided supervision and

For peer review only - http://bmjopen.bmj.com/site/about/guidelines.xhtml

guidance during the study. All authors reviewed and approved the manuscript in its current form. JMN is the guarantor of this work.

Funding Norris is funded by the Medical Research Council (MRC) (MR/S00680X/1). Simpson is funded by the Rosetrees Trust. Emberton receives research support from the United Kingdom's National Institute of Health Research (NIHR) UCLH/UCL Biochemical Research Centre.

Competing Interests Norris receives funding from the MRC. Simpson receives funding from the Rosetrees Trust. Whitaker receives funding from the PCUK, the Urology Foundation and Rosetrees Trust. Kirkham, Freeman and Emberton have stock interest in Nuada Medical Ltd. Emberton acts as a consultant, trainer and proctor to Sonatherm Inc; Angiodynamics Inc; Exact Imaging Inc.

Patient consent for publication Not required

Provenance and peer review Not commissioned; externally peer reviewed

Open access This is an open access article distributed in accordance with the Creative Commons Attribution 4.0 Unreported (CC BY 4.0) license which permits others to copy, redistribute, remix, transform and build upon this work for any purpose, provided the original work is properly cited, a link to the licence is given, and indication of whether changes were made. See https://creativecommons.org/licenses/by/4.0/.

ORCID IDs

Naomi Morka https://orcid.org/0000-0002-1925-7223

Joseph M. Norris http://orcid.org/0000-0003-2294-0303

Benjamin S. Simpson http://orcid.org/0000-0003-3685-6110

For peer review only - http://bmjopen.bmj.com/site/about/guidelines.xhtml

REFERENCES

1. Ferlay J, Ervik M, Lam F, et al. Global Cancer Observatory: Cancer Today. Lyon, France: International Agency for Research on Cancer. https://gco.iarc.fr/today. Published 2018. Accessed October 24, 2020.
2. Mertan F V., Greer MD, Borofsky S, et al. Multiparametric Magnetic Resonance Imaging of Recurrent Prostate Cancer. *Top Magn Reson Imaging*. 2016;25(3):139-147.
3. Tourinho-Barbosa R, Srougi V, Nunes-Silva I, et al. Biochemical recurrence after radical prostatectomy: what does it mean? *Int braz j urol*. 2018;44(1):14-21.
4. Cookson MS, Aus G, Burnett AL, et al. Variation in the Definition of Biochemical Recurrence in Patients Treated for Localized Prostate Cancer: The American Urological Association Prostate Guidelines for Localized Prostate Cancer Update Panel Report and Recommendations for a Standard in the Re. *J Urol*. 2007;177(2):540-545.
5. Loeb S, Zhu X, Schroder FH, Roobol MJ. Long-term radical prostatectomy outcomes among participants from the European Randomized Study of Screening for Prostate Cancer (ERSPC) Rotterdam. *BJU Int*. 2012;110(11):1678-1683.
6. Boorjian SA, Eastham JA, Graefen M, et al. A Critical Analysis of the Long-Term Impact of Radical Prostatectomy on Cancer Control and Function Outcomes. *Eur Urol*. 2012;61(4):664-675.
7. Van den Broeck T, van den Bergh RCN, Arfi N, et al. Prognostic Value of Biochemical Recurrence Following Treatment with Curative Intent for Prostate Cancer: A Systematic Review. *Eur Urol*. 2019;75(6):967-987.
8. Roberts WB, Han M. Clinical significance and treatment of biochemical recurrence after definitive therapy for localized prostate cancer. *Surg Oncol*. 2009;18(3):268-274.
9. Walz J, Chun FK-H, Klein EA, et al. Nomogram Predicting the Probability of Early Recurrence After Radical Prostatectomy for Prostate Cancer. *J Urol*. 2009;181(2):601-608.
10. Cooperberg MR, Hilton JF, Carroll PR. The CAPRA-S score. *Cancer*. 2011;117(22):5039-5046.
11. Stephenson AJ, Scardino PT, Eastham JA, et al. Postoperative Nomogram Predicting the 10-Year Probability of Prostate Cancer Recurrence After Radical Prostatectomy. *J Clin Oncol*. 2005;23(28):7005-7012.
12. D'Amico A V. Biochemical Outcome After Radical Prostatectomy, External Beam Radiation Therapy, or Interstitial Radiation Therapy for Clinically Localized Prostate Cancer. *JAMA*. 1998;280(11):969.

For peer review only - http://bmjopen.bmj.com/site/about/guidelines.xhtml

13. Zhao Z, Weickmann S, Jung M, et al. A Novel Predictor Tool of Biochemical Recurrence after Radical Prostatectomy Based on a Five-MicroRNA Tissue Signature. *Cancers (Basel)*. 2019;11(10):1603.

14. Remmers S, Verbeek JFM, Nieboer D, van der Kwast T, Roobol MJ. Predicting biochemical recurrence and prostate cancer-specific mortality after radical prostatectomy: comparison of six prediction models in a cohort of patients with screening- and clinically detected prostate cancer. *BJU Int*. 2019;124(4):635-642.

15. Martini A, Gupta A, Lewis SC, et al. Development and internal validation of a side-specific, multiparametric magnetic resonance imaging-based nomogram for the prediction of extracapsular extension of prostate cancer. *BJU Int*. 2018;122(6):1025-1033.

16. Gandaglia G, Ploussard G, Valerio M, et al. The Key Combined Value of Multiparametric Magnetic Resonance Imaging, and Magnetic Resonance Imaging–targeted and Concomitant Systematic Biopsies for the Prediction of Adverse Pathological Features in Prostate Cancer Patients Undergoing Radical Prostatect. *Eur Urol*. 2020;77(6):733-741.

17. Feng TS, Sharif-Afshar AR, Wu J, et al. Multiparametric MRI Improves Accuracy of Clinical Nomograms for Predicting Extracapsular Extension of Prostate Cancer. *Urology*. 2015;86(2):332-337.

18. Norris JM, Carmona Echeverria LM, Bott SRJ, et al. What Type of Prostate Cancer Is Systematically Overlooked by Multiparametric Magnetic Resonance Imaging? An Analysis from the PROMIS Cohort. *Eur Urol*. 2020;78(2):163-170.

19. Park JJ, Kim CK, Park SY, Park BK, Lee HM, Cho SW. Prostate Cancer: Role of Pretreatment Multiparametric 3-T MRI in Predicting Biochemical Recurrence After Radical Prostatectomy. *Am J Roentgenol*. 2014;202(5):W459-W465.

20. Shiradkar R, Ghose S, Jambor I, et al. Radiomic features from pretreatment biparametric MRI predict prostate cancer biochemical recurrence: Preliminary findings. *J Magn Reson Imaging*. 2018;48(6):1626-1636.

21. Li P, You S, Nguyen C, et al. Genes involved in prostate cancer progression determine MRI visibility. *Theranostics*. 2018;8(7):1752-1765.

22. Moher D, Shamseer L, Clarke M, et al. Preferred reporting items for systematic review and meta-analysis protocols (PRISMA-P) 2015 statement. *Syst Rev*. 2015;4(1):1.

For peer review only - http://bmjopen.bmj.com/site/about/guidelines.xhtml

23. Damadian R, Goldsmith M, Minkoff L. NMR in cancer: XVI. FONAR image of the live human body. *Physiol Chem Phys*. 1977;9(1):97-100, 108.

24. Norris JM, Simpson BS, Parry MA, et al. Genetic Landscape of Prostate Cancer Conspicuity on Multiparametric Magnetic Resonance Imaging: A Systematic Review and Bioinformatic Analysis. *Eur Urol Open Sci*. 2020;20(49):37-47.

25. O'Connor E, Simpson BS, Houlden H, Vandrovcova J, Matharu M. Prevalence of familial cluster headache: a systematic review and meta-analysis. *J Headache Pain*. 2020;21(1):37.

26. Blum DL, Koyama T, M'Koma AE, et al. Chemokine Markers Predict Biochemical Recurrence of Prostate Cancer following Prostatectomy. *Clin Cancer Res*. 2008;14(23):7790-7797.

27. Arumainayagam N, Ahmed HU, Moore CM, et al. Multiparametric MR Imaging for Detection of Clinically Significant Prostate Cancer: A Validation Cohort Study with Transperineal Template Prostate Mapping as the Reference Standard. *Radiology*. 2013;268(3):761-769.

28. Kim JY, Kim SH, Kim YH, Lee HJ, Kim MJ, Choi MS. Low-Risk Prostate Cancer: The Accuracy of Multiparametric MR Imaging for Detection. *Radiology*. 2014;271(2):435-444.

29. Rastinehad AR, Baccala AA, Chung PH, et al. D'Amico Risk Stratification Correlates With Degree of Suspicion of Prostate Cancer on Multiparametric Magnetic Resonance Imaging. *J Urol*. 2011;185(3):815-820.

30. Weinreb JC, Barentsz JO, Choyke PL, et al. PI-RADS Prostate Imaging - Reporting and Data System: 2015, Version 2. *Eur Urol*. 2016;69(1):16-40.

31. Zhang Y-D, Wu C-J, Bao M-L, et al. MR-based prognostic nomogram for prostate cancer after radical prostatectomy. *J Magn Reson Imaging*. 2017;45(2):586-596.

32. Park SY, Oh YT, Jung DC, et al. Prediction of biochemical recurrence after radical prostatectomy with PI-RADS version 2 in prostate cancers: initial results. *Eur Radiol*. 2016;26(8):2502-2509.

33. Hattori S, Kosaka T, Mizuno R, et al. Prognostic value of preoperative multiparametric magnetic resonance imaging (MRI) for predicting biochemical recurrence after radical prostatectomy. *BJU Int*. 2014;113(5):741-747.

For peer review only

Reporting checklist for protocol of a systematic review.

Based on the PRISMA-P guidelines.

Instructions to authors

Complete this checklist by entering the page numbers from your manuscript where readers will find each of the items listed below.

Your article may not currently address all the items on the checklist. Please modify your text to include the missing information. If you are certain that an item does not apply, please write "n/a" and provide a short explanation.

Upload your completed checklist as an extra file when you submit to a journal.

In your methods section, say that you used the PRISMA-Preporting guidelines, and cite them as:

Moher D, Shamseer L, Clarke M, Ghersi D, Liberati A, Petticrew M, Shekelle P, Stewart LA. Preferred Reporting Items for Systematic Review and Meta-Analysis Protocols (PRISMA-P) 2015 statement. Syst Rev. 2015;4(1):1.

		Reporting Item	Page Number
**Title**			
Identification	#1a	Identify the report as a protocol of a systematic review	1
Update	#1b	If the protocol is for an update of a previous systematic review, identify as such	N/A (It is not an update of a previous review)
**Registration**			
	#2	If registered, provide the name of the registry (such as PROSPERO) and registration number	2
**Authors**			
Contact	#3a	Provide name, institutional affiliation, e-mail address of all protocol authors; provide physical mailing address of corresponding author	1 and 10
Contribution	#3b	Describe contributions of protocol authors and identify the guarantor of the review	10

For peer review only

**Amendments**			
	#4	If the protocol represents an amendment of a previously completed or published protocol, identify as such and list changes; otherwise, state plan for documenting important protocol amendments	N/A (This protocol is not an amendment of a previous protocol)
**Support**			
Sources	#5a	Indicate sources of financial or other support for the review	1
Sponsor	#5b	Provide name for the review funder and / or sponsor	N/A (Review is not funded)
Role of sponsor or funder	#5c	Describe roles of funder(s), sponsor(s), and / or institution(s), if any, in developing the protocol	N/A (Review is not funded)
**Introduction**			
Rationale	#6	Describe the rationale for the review in the context of what is already known	4
Objectives	#7	Provide an explicit statement of the question(s) the review will address with reference to participants, interventions, comparators, and outcomes (PICO)	4
**Methods**			
Eligibility criteria	#8	Specify the study characteristics (such as PICO, study design, setting, time frame) and report characteristics (such as years considered, language, publication status) to be used as criteria for eligibility for the review	5
Information sources	#9	Describe all intended information sources (such as electronic databases, contact with study authors, trial registers or other grey literature sources) with planned dates of coverage	5
Search strategy	#10	Present draft of search strategy to be used for at least one electronic database, including planned limits, such that it could be repeated	10
Study records - data management	#11a	Describe the mechanism(s) that will be used to manage records and data throughout the review	5

For peer review only

Study records - selection process	#11b	State the process that will be used for selecting studies (such as two independent reviewers) through each phase of the review (that is, screening, eligibility and inclusion in meta-analysis)	5
Study records - data collection process	#11c	Describe planned method of extracting data from reports (such as piloting forms, done independently, in duplicate), any processes for obtaining and confirming data from investigators	5
Data items	#12	List and define all variables for which data will be sought (such as PICO items, funding sources), any pre-planned data assumptions and simplifications	6
Outcomes and prioritization	#13	List and define all outcomes for which data will be sought, including prioritization of main and additional outcomes, with rationale	7
Risk of bias in individual studies	#14	Describe anticipated methods for assessing risk of bias of individual studies, including whether this will be done at the outcome or study level, or both; state how this information will be used in data synthesis	7
Data synthesis	#15a	Describe criteria under which study data will be quantitatively synthesised	7
Data synthesis	#15b	If data are appropriate for quantitative synthesis, describe planned summary measures, methods of handling data and methods of combining data from studies, including any planned exploration of consistency (such as I2, Kendall's τ)	7
Data synthesis	#15c	Describe any proposed additional analyses (such as sensitivity or subgroup analyses, meta-regression)	7
Data synthesis	#15d	If quantitative synthesis is not appropriate, describe the type of summary planned	N/A (Quantitative synthesis is appropriate)
Meta-bias(es)	#16	Specify any planned assessment of meta-bias(es) (such as publication bias across studies, selective reporting within studies)	7
Confidence in	#17	Describe how the strength of the body of evidence will be	7

For peer review only

cumulative evidence	assessed (such as GRADE)

Notes:

- 1b: N/A (It is not an update of a previous review)
- 4: N/A (This protocol is not an amendment of a previous protocol)
- 5b: N/A (Review is not funded)
- 5c: N/A (Review is not funded)
- 15d: N/A (Quantitative synthesis is appropriate) The PRISMA-P checklist is distributed under the terms of the Creative Commons Attribution License CC-BY 4.0. This checklist was completed on 03. December 2020 using https://www.goodreports.org/, a tool made by the EQUATOR Network in collaboration with Penelope.ai

For peer review only

BMJ Open

Clinical outcomes associated with prostate cancer conspicuity on biparametric and multiparametric magnetic resonance imaging: a protocol for a systematic review and meta-analysis of biochemical recurrence post-radical prostatectomy.

Journal:	*BMJ Open*
Manuscript ID	bmjopen-2020-047664.R1
Article Type:	Protocol
Date Submitted by the Author:	15-Feb-2021
Complete List of Authors:	Morka, Naomi; University College London Medical School,<br>Simpson, Benjamin Scott; University College London, UCL Division of Surgery & Interventional Science<br>Ball, Rhys; University College London Hospitals NHS Foundation Trust, Department of Pathology<br>Freeman, Alex; University College Hospital London, Department of Histopathology<br>Kirkham, Alex; University College London Hospitals NHS Foundation Trust, Department of Radiology<br>Kelly, Daniel; Cardiff University, School of Healthcare Sciences, College of Biomedical and Life Sciences<br>Whitaker, Hayley; University College London, UCL Division of Surgery and Interventional Science<br>Emberton, Mark; University College London, Division of Surgery and Interventional Sciences; University College London Hospital, Department of Urology<br>Norris, Joseph; University College London, UCL Division of Surgery & Interventional Science
**Primary Subject Heading**:	Urology
Secondary Subject Heading:	Radiology and imaging
Keywords:	UROLOGY, Prostate disease < UROLOGY, Urological tumours < UROLOGY, Magnetic resonance imaging < RADIOLOGY & IMAGING

SCHOLARONE™
Manuscripts

I, the Submitting Author has the right to grant and does grant on behalf of all authors of the Work (as defined in the below author licence), an exclusive licence and/or a non-exclusive licence for contributions from authors who are: i) UK Crown employees; ii) where BMJ has agreed a CC-BY licence shall apply, and/or iii) in accordance with the terms applicable for US Federal Government officers or employees acting as part of their official duties; on a worldwide, perpetual, irrevocable, royalty-free basis to BMJ Publishing Group Ltd ("BMJ") its licensees and where the relevant Journal is co-owned by BMJ to the co-owners of the Journal, to publish the Work in this journal and any other BMJ products and to exploit all rights, as set out in our licence.

The Submitting Author accepts and understands that any supply made under these terms is made by BMJ to the Submitting Author unless you are acting as an employee on behalf of your employer or a postgraduate student of an affiliated institution which is paying any applicable article publishing charge ("APC") for Open Access articles. Where the Submitting Author wishes to make the Work available on an Open Access basis (and intends to pay the relevant APC), the terms of reuse of such Open Access shall be governed by a Creative Commons licence – details of these licences and which Creative Commons licence will apply to this Work are set out in our licence referred to above.

Other than as permitted in any relevant BMJ Author's Self Archiving Policies, I confirm this Work has not been accepted for publication elsewhere, is not being considered for publication elsewhere and does not duplicate material already published. I confirm all authors consent to publication of this Work and authorise the granting of this licence.

For peer review only

Clinical outcomes associated with prostate cancer conspicuity on biparametric and multiparametric magnetic resonance imaging: a protocol for a systematic review and meta-analysis of biochemical recurrence post-radical prostatectomy

Naomi Morka[1], Benjamin S. Simpson[2], Rhys Ball[3], Alex Freeman[3], Alex Kirkham[4], Daniel Kelly[5], Hayley C. Whitaker[2], Mark Emberton,[1,6]* Joseph M. Norris[2,6,7]

ME and JMN are joint senior authors.

[1] University College London Medical School, London, UK.

[2] UCL Division of Surgery & Interventional Science, University College London, London, UK

[3] Department of Pathology, University College London Hospitals NHS Foundation Trust, London, UK

[4] Department of Radiology, University College London Hospitals NHS Foundation Trust, London, UK

[5] School of Healthcare Sciences, Cardiff University, Wales, UK

[6] London Deanery of Urology, London, UK

[7] Department of Urology, University College London Hospitals NHS Foundation Trust, London, UK

Correspondence to: Joseph M Norris;

UCL Division of Surgery & Interventional Science

43-45 Foley St, Fitzrovia, London. W1W 7TY

joseph.norris@ucl.ac.uk

Funding: Norris is funded by the Medical Research Council (MRC) (MR/S00680X/1). Simpson is funded by the Rosetrees Trust. Emberton receives research support from the United Kingdom's National Institute of Health Research (NIHR) UCLH/UCL Biochemical Research Centre.

Ethics: No ethical considerations apply.

Word count: 1736 words

Conflicts of interest: Norris receives funding from the MRC. Simpson receives funding from the Rosetrees Trust. Whitaker receives funding from the PCUK, the Urology Foundation and Rosetrees Trust. Kirkham, Freeman and Emberton have stock interest in Nuada Medical Ltd. Emberton acts as a consultant, trainer and proctor to Sonatherm Inc; Angiodynamics Inc; Exact Imaging Inc.

For peer review only - http://bmjopen.bmj.com/site/about/guidelines.xhtml

ABSTRACT

Introduction: There is an increasing body of evidence to suggest that visibility of prostate cancer on magnetic resonance (MRI) may be related to likelihood of adverse pathological outcomes. Biochemical recurrence (BCR) after radical prostatectomy remains a significant clinical challenge and a means of predicting likelihood of this prior to surgery could inform treatment choice. It appears that MRI could be a potential candidate strategy for BCR prediction, and as such, there is a need to review extant literature on the prognostic capability of MRI. Here, we describe a protocol for a systematic review and meta-analysis of the utility of biparametric MRI (bpMRI) and multiparametric MRI (mpMRI) in predicting biochemical recurrence following radical prostatectomy for prostate cancer treatment.

Methods and analysis: PubMed, MEDLINE, Embase and Cochrane databases will be searched and screening will be guided by the preferred reporting items for systematic reviews and meta-analyses (PRISMA) guidelines. In order to meet the inclusion criteria, papers must be English-language articles involving patients who have had bpMRI or mpMRI for suspected prostate cancer and have undergone radical prostatectomy as definitive therapy. Patients must have had prostate specific antigen (PSA) monitoring before and after surgery. All relevant papers published from July 1977 to October 2020 will be eligible for inclusion. The Newcastle-Ottawa score will be used to determine the quality and bias of the studies. This protocol is written in-line with the PRISMA protocol 2015 checklist.

Ethics and dissemination: There are no relevant ethical concerns. Dissemination of this protocol will be via peer-reviewed journals as well as national and international conferences.

PROSPERO registration number CRD42020206074

For peer review only

Strengths and limitations of this study

- This study will be the first comprehensive systematic review and meta-analysis of the utility of bpMRI and mpMRI in the prediction of biochemical recurrence following radical prostatectomy, written in-line with PRISMA guidelines.
- This study will employ robust statistical methods to minimise the effect of heterogeneity in the literature on the determined results.
- Heterogeneity of study cohorts may limit the strength of conclusions drawn.
- Due to limited number of studies which make use of bpMRI, there is likely to be a less extensive analysis of the prognostic utility of this modality.

For peer review only - http://bmjopen.bmj.com/site/about/guidelines.xhtml

- Also, due to the relatively recent introduction of prostatic MRI scans, there is limited long term data on the clinical outcomes associated with MRI.

For peer review only

For peer review only - http://bmjopen.bmj.com/site/about/guidelines.xhtml

BACKGROUND

Prostate cancer is a one of the leading causes of cancer mortality in men worldwide, with over a million new cases diagnosed in 2018 alone[1]. Radical prostatectomy is the most commonly employed definitive treatment for patients with this disease[2], often yielding favourable oncologic outcomes[3]. However, biochemical recurrence (BCR), taken to be a serum prostate specific antigen (PSA) level greater than or equal to 0.2ng/dL on two consecutive occasions[4] occurs in approximately 15-40% of patients[5,6]. Prognostically, BCR has high utility and is associated with increased incidence of metastatic disease[4,7]. It follows then, that a reliable approach to predicting BCR could inform therapeutic decision making[8]. Several models have been proposed for prognostication of BCR following radical prostatectomy[9–12], however, these have been criticised for apparent inaccuracy and inapplicability to certain clinical settings[13,14]. This highlights the need for a widely acceptable and accessible means of understanding prostate cancer prognosis in this patient group.

Magnetic resonance imaging (MRI) has increasingly become an integral part of prostate cancer screening and diagnosis. In recent years, evidence has emerged on the utility of multiparametric MRI (mpMRI) in predicting adverse pathology with MRI-invisible tumours suggesting a good prognosis and MRI-visible tumours associated with unfavourable clinical outcomes such as extracapsular extension and seminal vesicle invasion[15–18]. Given that these pathological factors are also associated with increased likelihood of BCR[9,10], it has been suggested that mpMRI may have a role in the prediction of BCR after radical prostatectomy[19,20]. This is also supported by studies that show that the visibility of prostate tumours on mpMRI may corelate with genetic markers of poor clinical outcome[21] . Biparametric MRI (bpMRI) has been considered as a potential alternative to mpMRI[22]. Although its diagnostic utility is still being appraised, there is a growing interest in this modality as it has been suggested that it could reduce costs and imaging times[23,24]. Further exploration of the role of MRI in predicting BCR after radical prostatectomy is necessary as its use in this way could enhance and improve existing models of prostate cancer prognosis, allowing for better clinical decision making and patient care.

The aim of this systematic review is to evaluate and collate the existing literature on the prognostication of BCR after radical prostatectomy using pre-treatment bpMRI or mpMRI. This will be the first of its kind and it will assess the utility of pre-operative MRI as a risk stratification tool and predictor of long term clinical outcomes.

For peer review only - http://bmjopen.bmj.com/site/about/guidelines.xhtml

METHODS AND ANALYSIS

This protocol for this systematic review has been written in compliance with the guidance set out in the Preferred Reporting Items for Systematic Reviews and Meta-analysis Protocol (PRISMA-P) 2015 checklist[25]. Included studies will undergo thorough appraisal and analysis to reveal the extent to which pre-operative MRI status results may be indicative of the likelihood to develop biochemical recurrence after radical prostatectomy. This protocol has been registered on the PROSPERO database with the registration number: CRD42020206074.

Search Methodology

Searches will be performed on the PubMed, MEDLINE, Embase and Cochrane databases to identify all potentially relevant studies published from July 1977 (date of first human MRI)[26] to October 2020. Free text searches and Medical Subject Headings (MESH) terms will be used with suitable Boolean operators. The use of search terms such as 'prostate,' 'cancer,' 'bpMRI' and 'mpMRI,' along with alternative terms for 'biochemical recurrence,' 'radical prostatectomy' and 'prognosis' will ensure a comprehensive approach. Three reviewers will be involved in article selection and evaluation and will employ the use Rayyan, a systematic review collaboration tool to improve ease of collaboration, screening of studies and removal of duplicates. To minimize likelihood of overlooking relevant literature, reference lists for each article will be manually screened and experts in the field will be approached.

Study selection and data extraction

Studies will be screened by three separate reviewers using their title and abstract. Following this, the full text articles will then be scrutinized against the inclusion criteria. Any disagreements will be resolved by consensus. Exclusions will be documented with the reasons for each case, allowing generation of the PRISMA flowchart.

Inclusion criteria

To be selected for this review, studies must involve patients with suspected prostate cancer who have undergone a pre-treatment bpMRI or mpMRI, and both pre- and post-radical prostatectomy serum PSA testing. Only studies which use grades of conspicuity (e.g PI-RADS, Likert scale etc.) to assess the relationship between MRI visibility and biochemical recurrence will be included.

For peer review only - http://bmjopen.bmj.com/site/about/guidelines.xhtml

Exclusion criteria

Case reports, expert opinions, conference abstracts, reviews and non-English language articles will be ineligible for inclusion. Studies with patient cohorts who have received treatment prior to surgery will also be excluded.

Data extraction

After selection, all articles will meticulously read and examined, with relevant data points extracted and inputted onto a group spreadsheet. Three or more independent reviewers will confirm the data and check for accuracy. Collation of data will be carried out as described by our group previously[27]. The data to be collected is summarised in Table 1.

Table 1 **Data collection items**

**Item number**	Data title	Data type
**1**	Year of publication	Study characteristic
**2**	Author names	Study characteristic
**3**	Study design	Study characteristic
**4**	Patient population	Demographics
**5**	Number of patients	Demographics
**6**	Pre-operative MRI status	Demographics
**7**	Number of years of radiologist experience	Methodology
**8**	MRI scoring system	Methodology
**9**	MRI scanner power	Methodology
**10**	Definition of tumour visibility	Methodology
**11**	Definition of biochemical recurrence	Methodology
**12**	Total follow up time	Methodology
**13**	Biochemical recurrence status	Outcome
**14**	Hazard ratio of BCR	Outcome

BCR, biochemical recurrence; MRI; magnetic resonance imaging; PSA, prostate specific antigen.

For peer review only - http://bmjopen.bmj.com/site/about/guidelines.xhtml

For peer review only

Endpoints

The main endpoint of this review will be the identification of significant differences in effect estimates such as hazard ratios for BCR between MRI-visible (e.g PI-RADS 3-5) and MRI-invisible (e.g PI-RADS 1-2) groups. Cancer-specific and all-cause mortality will be the secondary endpoints. Moderator variables, such as MRI scoring systems (e.g. PI-RADS vs Likert) and MRI scanner power will be compared, and any which are of a notable impact to the final summary estimate will be recorded.

Risk of bias in individual studies

The bias and quality of the included studies will be assessed by three independent reviewers using the Newcastle-Ottawa score. The three sections of this system (selection, comparability and outcome) will allow for an evaluation of the methods and assessment of the quality of each study. The outcome of which will aid in the thematic synthesis. Any disagreements between the reviewers will be resolved by consensus. In the case that a study is found to be at risk of significant bias or is of unsatisfactory standard, it may be excluded. If the study is still deemed fit to be included in the analysis, this will be duly noted as a limitation in the discussion and conclusion portions of the systematic review. As previously described by us elsewhere[28], sections of the Newcastle-Ottawa scoring system may be modified to better suit the subject area of this review and ensure a greater accuracy in the findings. Risk of bias score will be assessed as a potential moderator variable in the meta-analysis.

Meta-analysis

Hazard ratios of BCR from univariate Cox regression analyses of MRI visibility groups will be used to create the summary estimate for the meta-analysis. Studies which have incorporated hazard ratios for MRI visibility into multivariable models will not be included in the metanalysis unless the univariate data is available. The distribution of untransformed, logit and double-arcsine transformed ratios will be compared and will be assessed for normality using density plots and tested using the Shapiro-Wilk test. Whichever set of ratios most resemble a normal distribution will be used for further analysis. Inter-study variation will be quantified using I^2 and a random-effects model will be fitted for estimation the summary estimate. After fitting a model to all relevant studies, leave-one-out analyses (LOO) and accompanying diagnostic plots would be used to identify influential studies including: externally studentized residuals, difference in fits values (DFFITS),

For peer review only - http://bmjopen.bmj.com/site/about/guidelines.xhtml

Cook's distances, covariance ratios, LOO estimates of the amount of heterogeneity, LOO values of the test statistics for heterogeneity, hat values and weights. Each study would be removed one at a time, and the summary proportion is re-estimated based on the remaining n-1 studies. Studies with a statistically significant influence on the fitted model would be removed as outliers and the model re-fitted. Finally, a summary estimate comprising the remaining studies will be calculated to estimate the true hazard associated with MRI visibility or the inverse, a MRI invisibility. Sub-group analyses are planned between Likert and PI-RADS scoring systems. Data collection items 1,4,7,8,9,10,11 and 12 will be tested as potential moderators assuming a sufficient number of studies are present within each group. All data analysis and visualisation will be performed using the R statistical environment (version 3.6.1, 2019-07-05) using the "metafor" and "meta" packages.

Patient and public involvement

No patients were involved in this study.

For peer review only

DISCUSSION

There is an increasing need to risk stratify prostate cancers, to accurately identify disease that is more likely to take an adverse clinical course[1,29]. This systematic review aims to address this by appraising current evidence on whether tumour MRI visibility is related to BCR of prostate cancer after radical prostatectomy, therefore evaluating the potential use of bpMRI and mpMRI as risk stratification tools in this population.

Current evidence suggests an association between visibility of prostate cancer on MRI with Gleason grade and tumour size[30,31]. Furthermore, it appears that MRI conspicuity is positively correlated with D'Amico risk nomogram scores[32]. In terms of the prognostic potential of MRI, it has been demonstrated that predictive power of MRI may extend beyond its relationship with histopathological markers of disease progression. Indeed, raised Prostate Imaging-Reporting and Data System (PI-RADS) scores[33] and apparent diffusion coefficient (ADC) values have been shown to independently predict the likelihood of BCR[34,35] .

Although there is a clear potential role for bpMRI and mpMRI in predicting BCR after radical prostatectomy, across studies, there are variations in methodology, such as biopsy sampling, definitions of MRI positivity and clinically significant prostate cancer. This, together with the unavailability of extensive long-term data on MRI outcomes may create a limitation in the ability to form robust conclusions in this review. Also, due to inexhaustive reporting of tumour characteristics, there is often a lack of data on features such as tumour volume and clinical stage within MRI-invisible and MRI-visible groups. This poses a potential difficultly in understanding the effects that these factors may have on the determined results of the meta-analysis. We hope to address the heterogeneity in the literature and encourage a more uniform approach to research in this subject area.

In summary, early evidence suggests that bpMRI and mpMRI could provide a means to predict BCR of prostate cancer after radical prostatectomy. The wide availability of MRI and its non-invasive nature makes it an ideal candidate for potential BCR risk stratification[36]. Our systematic review will assess and analyse the available evidence, with the aim of providing a holistic view of the literature. This will lead to a clearer understanding of the subject, which could inform the choice of appropriate therapy in patients with prostate cancer.

For peer review only - http://bmjopen.bmj.com/site/about/guidelines.xhtml

Trial status

- Preliminary searches: started.
- Piloting of the selection study process: not started.
- Formal screening: not started.
- Data extraction: not started.
- Risk of bias assessment: not started.
- Data analysis: not started.

Draft of search strategy for MEDLINE, Embase, PubMed and Cochrane Databases: ((prostat* NOT prostatitis) AND ("cancer" OR tumo?r* OR malignancy*)) AND ("magnetic resonance imaging" OR "MRI" OR "mpMRI" OR "multi-parametric magnetic resonance imaging" OR "bpMRI" OR "bi-parametric magnetic resonance imaging") AND ("biochemical recurrence" OR "BCR" OR "biochemical failure") AND ("radical prostatectomy" OR "surgery") AND (prognos* OR predict*)

ETHICS AND DISSEMINATION

There are no relevant ethical concerns. Dissemination of this protocol will be via peer-reviewed journals as well as national and international conferences.

Author affiliations

[1] University College London Medical School, London, UK.

[2] UCL Division of Surgery & Interventional Science, University College London, London, UK

[3] Department of Pathology, University College London Hospitals NHS Foundation Trust, London, UK

[4] Department of Radiology, University College London Hospitals NHS Foundation Trust, London, UK

[5] School of Healthcare Sciences, Cardiff University, Wales, UK

[6] London Deanery of Urology, London, UK

[7] Department of Urology, University College London Hospitals NHS Foundation Trust, London, UK

Email Naomi Morka: naomi.morka.17@ucl.ac.uk, Benjamin S. Simpson: b.simpson@ucl.ac.uk,

For peer review only - http://bmjopen.bmj.com/site/about/guidelines.xhtml

Rhys Ball: rhys.ball@nhs.net, Alex Freeman: alex.freeman2@nhs.net, Alex Kirkam: alexkirkham@nhs.net, Daniel Kelly: kellydm@cardiff.ac.uk, Hayley C. Whitaker: hayley.whitaker@ucl.ac.uk, Mark Emberton: m.emberton@ucl.ac.uk, Joseph M. Norris: joseph.norris@ucl.ac.uk

Twitter Naomi Morka @naomi_morka, Benjamin S. Simpson @b3nsimp, Daniel Kelly @profdkelly, Hayley C. Whitaker @HayleyWhitaker, Mark Emberton @EmbertonMark, Joseph M. Norris @MrJosephNorris

Contributors The authors' contribution includes, but is not limited to, the following: NM, BSS and JMN drafted the manuscript and created the study concept. RB, AK, AF, DK, HCW and ME provided supervision and guidance during the study. All authors reviewed and approved the manuscript in its current form. JMN is the guarantor of this work.

Funding Norris is funded by the Medical Research Council (MRC) (MR/S00680X/1). Simpson is funded by the Rosetrees Trust. Emberton receives research support from the United Kingdom's National Institute of Health Research (NIHR) UCLH/UCL Biochemical Research Centre.

Competing Interests Norris receives funding from the MRC. Simpson receives funding from the Rosetrees Trust. Whitaker receives funding from the PCUK, the Urology Foundation and Rosetrees Trust. Kirkham, Freeman and Emberton have stock interest in Nuada Medical Ltd. Emberton acts as a consultant, trainer and proctor to Sonatherm Inc; Angiodynamics Inc; Exact Imaging Inc.

Patient consent for publication Not required

Provenance and peer review Not commissioned; externally peer reviewed

Open access This is an open access article distributed in accordance with the Creative Commons Attribution 4.0 Unreported (CC BY 4.0) license which permits others to copy, redistribute, remix, transform and build upon this work for any purpose, provided the original work is properly cited, a link to the licence is given, and indication of whether changes were made. See https://creativecommons.org/licenses/by/4.0/.

For peer review only

For peer review only - http://bmjopen.bmj.com/site/about/guidelines.xhtml

ORCID IDs

Naomi Morka https://orcid.org/0000-0002-1925-7223

Joseph M. Norris http://orcid.org/0000-0003-2294-0303

Benjamin S. Simpson http://orcid.org/0000-0003-3685-6110

For peer review only

REFERENCES

1. Ferlay J, Ervik M, Lam F, et al. Global Cancer Observatory: Cancer Today. Lyon, France: International Agency for Research on Cancer. Published 2018. Accessed October 24, 2020. https://gco.iarc.fr/today
2. Mertan F V., Greer MD, Borofsky S, et al. Multiparametric Magnetic Resonance Imaging of Recurrent Prostate Cancer. *Top Magn Reson Imaging*. 2016;25(3):139-147. doi:10.1097/RMR.0000000000000088
3. Tourinho-Barbosa R, Srougi V, Nunes-Silva I, et al. Biochemical recurrence after radical prostatectomy: what does it mean? *Int braz j urol*. 2018;44(1):14-21. doi:10.1590/s1677-5538.ibju.2016.0656
4. Cookson MS, Aus G, Burnett AL, et al. Variation in the Definition of Biochemical Recurrence in Patients Treated for Localized Prostate Cancer: The American Urological Association Prostate Guidelines for Localized Prostate Cancer Update Panel Report and Recommendations for a Standard in the Re. *J Urol*. 2007;177(2):540-545. doi:10.1016/j.juro.2006.10.097
5. Loeb S, Zhu X, Schroder FH, Roobol MJ. Long-term radical prostatectomy outcomes among participants from the European Randomized Study of Screening for Prostate Cancer (ERSPC) Rotterdam. *BJU Int*. 2012;110(11):1678-1683. doi:10.1111/j.1464-410X.2012.11367.x
6. Boorjian SA, Eastham JA, Graefen M, et al. A Critical Analysis of the Long-Term Impact of Radical Prostatectomy on Cancer Control and Function Outcomes. *Eur Urol*. 2012;61(4):664-675. doi:10.1016/j.eururo.2011.11.053
7. Van den Broeck T, van den Bergh RCN, Arfi N, et al. Prognostic Value of Biochemical Recurrence Following Treatment with Curative Intent for Prostate Cancer: A Systematic Review. *Eur Urol*. 2019;75(6):967-987. doi:10.1016/j.eururo.2018.10.011
8. Roberts WB, Han M. Clinical significance and treatment of biochemical recurrence after definitive therapy for localized prostate cancer. *Surg Oncol*. 2009;18(3):268-274. doi:10.1016/j.suronc.2009.02.004
9. Walz J, Chun FK-H, Klein EA, et al. Nomogram Predicting the Probability of Early Recurrence After Radical Prostatectomy for Prostate Cancer. *J Urol*. 2009;181(2):601-608. doi:10.1016/j.juro.2008.10.033
10. Cooperberg MR, Hilton JF, Carroll PR. The CAPRA-S score. *Cancer*. 2011;117(22):5039-5046.

For peer review only - http://bmjopen.bmj.com/site/about/guidelines.xhtml

doi:10.1002/cncr.26169

11. Stephenson AJ, Scardino PT, Eastham JA, et al. Postoperative Nomogram Predicting the 10-Year Probability of Prostate Cancer Recurrence After Radical Prostatectomy. *J Clin Oncol*. 2005;23(28):7005-7012. doi:10.1200/JCO.2005.01.867
12. D'Amico A V. Biochemical Outcome After Radical Prostatectomy, External Beam Radiation Therapy, or Interstitial Radiation Therapy for Clinically Localized Prostate Cancer. *JAMA*. 1998;280(11):969. doi:10.1001/jama.280.11.969
13. Zhao Z, Weickmann S, Jung M, et al. A Novel Predictor Tool of Biochemical Recurrence after Radical Prostatectomy Based on a Five-MicroRNA Tissue Signature. *Cancers (Basel)*. 2019;11(10):1603. doi:10.3390/cancers11101603
14. Remmers S, Verbeek JFM, Nieboer D, van der Kwast T, Roobol MJ. Predicting biochemical recurrence and prostate cancer-specific mortality after radical prostatectomy: comparison of six prediction models in a cohort of patients with screening- and clinically detected prostate cancer. *BJU Int*. 2019;124(4):635-642. doi:10.1111/bju.14790
15. Martini A, Gupta A, Lewis SC, et al. Development and internal validation of a side-specific, multiparametric magnetic resonance imaging-based nomogram for the prediction of extracapsular extension of prostate cancer. *BJU Int*. 2018;122(6):1025-1033. doi:10.1111/bju.14353
16. Gandaglia G, Ploussard G, Valerio M, et al. The Key Combined Value of Multiparametric Magnetic Resonance Imaging, and Magnetic Resonance Imaging–targeted and Concomitant Systematic Biopsies for the Prediction of Adverse Pathological Features in Prostate Cancer Patients Undergoing Radical Prostatect. *Eur Urol*. 2020;77(6):733-741. doi:10.1016/j.eururo.2019.09.005
17. Feng TS, Sharif-Afshar AR, Wu J, et al. Multiparametric MRI Improves Accuracy of Clinical Nomograms for Predicting Extracapsular Extension of Prostate Cancer. *Urology*. 2015;86(2):332-337. doi:10.1016/j.urology.2015.06.003
18. Norris JM, Carmona Echeverria LM, Bott SRJ, et al. What Type of Prostate Cancer Is Systematically Overlooked by Multiparametric Magnetic Resonance Imaging? An Analysis from the PROMIS Cohort. *Eur Urol*. 2020;78(2):163-170. doi:10.1016/j.eururo.2020.04.029
19. Park JJ, Kim CK, Park SY, Park BK, Lee HM, Cho SW. Prostate Cancer: Role of Pretreatment Multiparametric 3-T MRI in Predicting Biochemical Recurrence After Radical Prostatectomy. *Am J Roentgenol*. 2014;202(5):W459-W465. doi:10.2214/AJR.13.11381

For peer review only - http://bmjopen.bmj.com/site/about/guidelines.xhtml

20. Shiradkar R, Ghose S, Jambor I, et al. Radiomic features from pretreatment biparametric MRI predict prostate cancer biochemical recurrence: Preliminary findings. *J Magn Reson Imaging*. 2018;48(6):1626-1636. doi:10.1002/jmri.26178

21. Li P, You S, Nguyen C, et al. Genes involved in prostate cancer progression determine MRI visibility. *Theranostics*. 2018;8(7):1752-1765. doi:10.7150/thno.23180

22. Bosaily AE-S, Frangou E, Ahmed HU, et al. Additional Value of Dynamic Contrast-enhanced Sequences in Multiparametric Prostate Magnetic Resonance Imaging: Data from the PROMIS Study. *Eur Urol*. 2020;78(4):503-511. doi:10.1016/j.eururo.2020.03.002

23. Eldred-Evans D, Burak P, Connor MJ, et al. Population-Based Prostate Cancer Screening With Magnetic Resonance Imaging or Ultrasonography. *JAMA Oncol*. Published online February 11, 2021. doi:10.1001/jamaoncol.2020.7456

24. van der Leest M, Israël B, Cornel EB, et al. High Diagnostic Performance of Short Magnetic Resonance Imaging Protocols for Prostate Cancer Detection in Biopsy-naïve Men: The Next Step in Magnetic Resonance Imaging Accessibility. *Eur Urol*. 2019;76(5):574-581. doi:10.1016/j.eururo.2019.05.029

25. Moher D, Shamseer L, Clarke M, et al. Preferred reporting items for systematic review and meta-analysis protocols (PRISMA-P) 2015 statement. *Syst Rev*. 2015;4(1):1. doi:10.1186/2046-4053-4-1

26. Damadian R, Goldsmith M, Minkoff L. NMR in cancer: XVI. FONAR image of the live human body. *Physiol Chem Phys*. 1977;9(1):97-100, 108. http://www.ncbi.nlm.nih.gov/pubmed/909957

27. Norris JM, Simpson BS, Parry MA, et al. Genetic Landscape of Prostate Cancer Conspicuity on Multiparametric Magnetic Resonance Imaging: A Systematic Review and Bioinformatic Analysis. *Eur Urol Open Sci*. 2020;20(49):37-47. doi:10.1016/j.euros.2020.06.006

28. O'Connor E, Simpson BS, Houlden H, Vandrovcova J, Matharu M. Prevalence of familial cluster headache: a systematic review and meta-analysis. *J Headache Pain*. 2020;21(1):37. doi:10.1186/s10194-020-01101-w

29. Blum DL, Koyama T, M'Koma AE, et al. Chemokine Markers Predict Biochemical Recurrence of Prostate Cancer following Prostatectomy. *Clin Cancer Res*. 2008;14(23):7790-7797. doi:10.1158/1078-0432.CCR-08-1716

30. Arumainayagam N, Ahmed HU, Moore CM, et al. Multiparametric MR Imaging for Detection of Clinically Significant Prostate Cancer: A Validation Cohort Study with Transperineal Template

For peer review only - http://bmjopen.bmj.com/site/about/guidelines.xhtml

Prostate Mapping as the Reference Standard. *Radiology*. 2013;268(3):761-769. doi:10.1148/radiol.13120641

31. Kim JY, Kim SH, Kim YH, Lee HJ, Kim MJ, Choi MS. Low-Risk Prostate Cancer: The Accuracy of Multiparametric MR Imaging for Detection. *Radiology*. 2014;271(2):435-444. doi:10.1148/radiol.13130801

32. Rastinehad AR, Baccala AA, Chung PH, et al. D'Amico Risk Stratification Correlates With Degree of Suspicion of Prostate Cancer on Multiparametric Magnetic Resonance Imaging. *J Urol*. 2011;185(3):815-820. doi:10.1016/j.juro.2010.10.076

33. Weinreb JC, Barentsz JO, Choyke PL, et al. PI-RADS Prostate Imaging - Reporting and Data System: 2015, Version 2. *Eur Urol*. 2016;69(1):16-40. doi:10.1016/j.eururo.2015.08.052

34. Zhang Y-D, Wu C-J, Bao M-L, et al. MR-based prognostic nomogram for prostate cancer after radical prostatectomy. *J Magn Reson Imaging*. 2017;45(2):586-596. doi:10.1002/jmri.25441

35. Park SY, Oh YT, Jung DC, et al. Prediction of biochemical recurrence after radical prostatectomy with PI-RADS version 2 in prostate cancers: initial results. *Eur Radiol*. 2016;26(8):2502-2509. doi:10.1007/s00330-015-4077-5

36. Hattori S, Kosaka T, Mizuno R, et al. Prognostic value of preoperative multiparametric magnetic resonance imaging (MRI) for predicting biochemical recurrence after radical prostatectomy. *BJU Int*. 2014;113(5):741-747. doi:10.1111/bju.12329

For peer review only

Reporting checklist for protocol of a systematic review.

Based on the PRISMA-P guidelines.

Instructions to authors

Complete this checklist by entering the page numbers from your manuscript where readers will find each of the items listed below.

Your article may not currently address all the items on the checklist. Please modify your text to include the missing information. If you are certain that an item does not apply, please write "n/a" and provide a short explanation.

Upload your completed checklist as an extra file when you submit to a journal.

In your methods section, say that you used the PRISMA-Preporting guidelines, and cite them as:

Moher D, Shamseer L, Clarke M, Ghersi D, Liberati A, Petticrew M, Shekelle P, Stewart LA. Preferred Reporting Items for Systematic Review and Meta-Analysis Protocols (PRISMA-P) 2015 statement. Syst Rev. 2015;4(1):1.

		Reporting Item	Page Number
**Title**			
Identification	#1a	Identify the report as a protocol of a systematic review	1
Update	#1b	If the protocol is for an update of a previous systematic review, identify as such	N/A (It is not an update of a previous review)
**Registration**			
	#2	If registered, provide the name of the registry (such as PROSPERO) and registration number	2
**Authors**			
Contact	#3a	Provide name, institutional affiliation, e-mail address of all protocol authors; provide physical mailing address of corresponding author	1 and 10
Contribution	#3b	Describe contributions of protocol authors and identify the guarantor of the review	10

For peer review only

**Amendments**			
	#4	If the protocol represents an amendment of a previously completed or published protocol, identify as such and list changes; otherwise, state plan for documenting important protocol amendments	N/A (This protocol is not an amendment of a previous protocol)
**Support**			
Sources	#5a	Indicate sources of financial or other support for the review	1
Sponsor	#5b	Provide name for the review funder and / or sponsor	N/A (Review is not funded)
Role of sponsor or funder	#5c	Describe roles of funder(s), sponsor(s), and / or institution(s), if any, in developing the protocol	N/A (Review is not funded)
**Introduction**			
Rationale	#6	Describe the rationale for the review in the context of what is already known	4
Objectives	#7	Provide an explicit statement of the question(s) the review will address with reference to participants, interventions, comparators, and outcomes (PICO)	4
**Methods**			
Eligibility criteria	#8	Specify the study characteristics (such as PICO, study design, setting, time frame) and report characteristics (such as years considered, language, publication status) to be used as criteria for eligibility for the review	5
Information sources	#9	Describe all intended information sources (such as electronic databases, contact with study authors, trial registers or other grey literature sources) with planned dates of coverage	5
Search strategy	#10	Present draft of search strategy to be used for at least one electronic database, including planned limits, such that it could be repeated	10
Study records - data management	#11a	Describe the mechanism(s) that will be used to manage records and data throughout the review	5

For peer review only

Study records - selection process	#11b	State the process that will be used for selecting studies (such as two independent reviewers) through each phase of the review (that is, screening, eligibility and inclusion in meta-analysis)	5
Study records - data collection process	#11c	Describe planned method of extracting data from reports (such as piloting forms, done independently, in duplicate), any processes for obtaining and confirming data from investigators	5
Data items	#12	List and define all variables for which data will be sought (such as PICO items, funding sources), any pre-planned data assumptions and simplifications	6
Outcomes and prioritization	#13	List and define all outcomes for which data will be sought, including prioritization of main and additional outcomes, with rationale	7
Risk of bias in individual studies	#14	Describe anticipated methods for assessing risk of bias of individual studies, including whether this will be done at the outcome or study level, or both; state how this information will be used in data synthesis	7
Data synthesis	#15a	Describe criteria under which study data will be quantitatively synthesised	7
Data synthesis	#15b	If data are appropriate for quantitative synthesis, describe planned summary measures, methods of handling data and methods of combining data from studies, including any planned exploration of consistency (such as I2, Kendall's τ)	7
Data synthesis	#15c	Describe any proposed additional analyses (such as sensitivity or subgroup analyses, meta-regression)	7
Data synthesis	#15d	If quantitative synthesis is not appropriate, describe the type of summary planned	N/A (Quantitative synthesis is appropriate)
Meta-bias(es)	#16	Specify any planned assessment of meta-bias(es) (such as publication bias across studies, selective reporting within studies)	7
Confidence in	#17	Describe how the strength of the body of evidence will be	7

For peer review only

cumulative evidence	assessed (such as GRADE)

Notes:

- 1b: N/A (It is not an update of a previous review)
- 4: N/A (This protocol is not an amendment of a previous protocol)
- 5b: N/A (Review is not funded)
- 5c: N/A (Review is not funded)
- 15d: N/A (Quantitative synthesis is appropriate) The PRISMA-P checklist is distributed under the terms of the Creative Commons Attribution License CC-BY 4.0. This checklist was completed on 03. December 2020 using https://www.goodreports.org/, a tool made by the EQUATOR Network in collaboration with Penelope.ai

For peer review only
